# Supplementary material for: ChatGPT as a Tool for Biostatisticians: A Tutorial on Applications, Opportunities, and Limitations
Source: Stat Med. 2025 Oct 23;44(23-24):e70263. doi: 10.1002/sim.70263 (PMC12548020; doi:10.1002/sim.70263)
Supplement: Supplementary file 1 — Data S1. Supporting Information. [file SIM-44-0-s001.zip › Supplementary Materials Additional.zip!/3.5_Causal_Inferece/3.5_Causal_Chat2.pdf]

OpenAI

Combining statistical and causal thinking_2 - Biostatistics Expert Assistance

Please clear memory.

I've cleared the memory of our previous conversations. If there's anything new you'd like to work on or discuss, just let me know!

Take the role of an expert biostatistician for this Chat.

Understood — I'll take the role of an expert biostatistician. Let me know what you're working on or how I can assist you.

I have data from a randomised clinical trial. I want to compare the efficacy of the experimental treatment with that of the control on overall survival in patients with non-small cell lung cancer. In this two-arm RCT, a considerable number of patients in the control arm switched to the experimental treatment after disease progression, while those in the experimental arm never switched.
My goal is to estimate the causal effect of sustained intake of the experimental treatment versus sustained intake of control on Overall survival, had the control group patients not been allowed to switch. Based on the information provided above, please define my target Estimand, following the guidance from the Estimand framework (ICH-E9 addendum).

Based on your description and aligned with the **ICH E9(R1) addendum** on estimands, your goal is to estimate the **causal effect of sustained treatment with the experimental intervention versus sustained control** on **overall survival**, in a setting where **treatment switching in the control arm is considered an intercurrent event (ICE)**. Here's a formal definition of your target estimand:

Target Estimand (following ICH E9(R1) framework)

Attribute	Specification
**Treatment**	Sustained treatment with the experimental therapy versus sustained treatment with control (i.e., standard of care or placebo).
**Population**	All randomised patients with non-small cell lung cancer as per the inclusion/exclusion criteria of the trial.
**Variable (Endpoint)**	Overall Survival (OS), defined as the time from randomisation to death from any cause.

Attribute	Specification
**Intercurrent Event**	**Treatment switching** from control to experimental therapy following disease progression (applies only in the control arm).
**Handling Strategy**	**Hypothetical strategy**: estimate what OS would have been *had control patients not switched* to the experimental treatment.
**Summary Measure**	Difference in median OS or hazard ratio (HR) from a hypothetical contrast; estimation typically via causal methods (e.g., IPCW, RPSFT, or g-methods) to adjust for treatment switching.

Interpretation of the Estimand

This estimand aims to answer the following question:

> *"What would the difference in overall survival have been between the experimental and control arms if all patients had received their initially assigned treatment throughout follow-up, and no switching had occurred?"*

This approach allows for a **causal interpretation** of treatment efficacy under a **hypothetical scenario** that eliminates the confounding effect of post-progression switching.

Would you like help selecting or implementing the appropriate statistical method to estimate this estimand, such as IPCW, g-formula, or structural models like RPSFT?

The switching behaviour is affected by a set of different baseline and time-varying covariates. Here, I want to assume that the time-varying confounders that influence the switching behaviour and Overall survival are also themselves affected by prior treatment intake.
For e.g. ECOG score might not only affect whether patients should switch treatment or not, but its value might also be affected by prior treatment intake reflected by the switching behaviour.
In this case, the analytical problem becomes more complex. Can you explain?

Yes — your scenario introduces **treatment-confounder feedback**, which substantially complicates estimation of the causal effect of sustained treatment strategies. Here's a structured explanation tailored to your context:

---

1. Nature of the Problem

You're describing a setting where:

- **Treatment switching** (in the control arm) occurs after disease progression.
- **Switching decisions** depend on **time-varying covariates** (e.g., ECOG score, lesion size, progression status).
- These **time-varying covariates are themselves affected by prior treatment intake**.

This creates a feedback loop:

- Prior treatment → changes covariates (e.g., ECOG) → affects probability of future treatment switching → affects survival.

This is called **treatment-confounder feedback**.

---

2. Why This is a Problem

Standard methods (like a time-dependent Cox model or naive inverse probability of censoring weighting [IPCW]) fail because:

- **Time-varying confounders** (e.g., ECOG, progression) are **both mediators and confounders**.
  - As confounders: they affect future treatment decisions and outcomes.
  - As mediators: they lie on the causal pathway from earlier treatment to the outcome.
- **Adjusting for them using standard regression or naive IPCW would block part of the treatment effect**, leading to **biased estimation**.

---

3. Proper Analytical Approach

To correctly estimate the causal effect under sustained treatment strategies, you need **causal inference methods that handle treatment-confounder feedback**:

a) G-computation / Parametric G-formula

- Models the full joint distribution of treatment, time-varying covariates, and outcome.
- Simulates survival under sustained treatment regimes.

b) G-estimation of Structural Nested Models

- Estimates parameters of models where the treatment effect is parameterised conditional on the observed history.
- Handles feedback between treatment and covariates explicitly.

c) IPCW (Advanced)

- Can be adapted if used within **marginal structural models (MSMs)**.
- Requires correct modelling of **treatment and censoring weights**, accounting for time-varying covariates **affected by prior treatment**.

  Note: You must **not** simply adjust for post-treatment variables in regression — that would induce **collider bias or block mediation pathways**.

---

4. Causal DAG Illustration (Conceptually)

Your DAG likely includes:

- Baseline covariates L_0
- Time-varying covariates L_t (e.g., ECOG, progression)
- Treatment process A_t (switching = 1)

- Outcome Y (overall survival)

The arrows look like:

- $A_t \to L_{t+1} \to A_{t+1}$
- $L_t \to A_t$ and $L_t \to Y$
- $A_t \to Y$

This structure confirms treatment-confounder feedback.

---

5. Summary

You are facing a classic case of **dynamic treatment regimes** with **time-varying confounding affected by prior treatment**. Naive approaches won't suffice.

To validly estimate your **hypothetical estimand**, you must use **G-methods**, such as:

- The **parametric g-formula**,
- **Marginal structural models (MSMs)** estimated via **IPCW**, or
- **Structural nested accelerated failure time models (SNAFTMs)**.

Would you like to discuss which method may be most feasible for your data and assumptions?

Depict our research problem in a suitable DAG

Here is a **Directed Acyclic Graph (DAG)** representing your research problem — a randomised trial with **treatment switching after progression**, **time-varying confounding**, and **treatment-confounder feedback**.

Key Variables

- A_0: Randomised treatment (experimental vs control)
- L_0: Baseline covariates (e.g. age, ECOG at baseline)
- L_1: Time-varying covariates (e.g. progression status, ECOG post-baseline)
- A_1: Post-progression treatment (switching in the control arm)
- Y: Overall survival

DAG Structure (Textual Layout)

```nginx
L0    →    A0    →    L1    →    A1    →    Y
↓               ↓          ↓          ↓
→───────────→─────────→─────────→→
```

Explanation of Arrows

- $L_0 \rightarrow A_0$: Baseline covariates may affect randomisation (e.g., via stratification), though in an RCT, this is typically blocked by design.
- $A_0 \rightarrow L_1$: Initial treatment affects time-varying covariates (treatment impacts ECOG, tumour burden, etc.).
- $L_1 \rightarrow A_1$: Switching decisions depend on progression, ECOG, etc.
- $A_1 \rightarrow Y$: Switching influences survival.
- $L_0 \rightarrow L_1$: Baseline variables affect post-baseline clinical status.
- $L_1 \rightarrow Y$: Clinical status after progression affects survival.
- $A_0 \rightarrow Y$: Initial treatment directly affects survival.
- $A_0 \rightarrow A_1$: Only patients in the control arm can switch.

Key Feature: Treatment-Confounder Feedback

- $A_0 \rightarrow L_1 \rightarrow A_1$: Prior treatment affects covariates, which influence switching decisions.
- L_1 is **both a confounder of $A_1 \rightarrow Y$** and a **mediator of $A_0 \rightarrow Y$** — making standard adjustment invalid.

Would you like me to generate a **graphical DAG image** of this?

I would like to apply the inverse probability of censoring weighting (IPCW) approach to address the bias introduced by switching following the methodology discussed in the following paper: Robins JM, Finkelstein DM. Correcting for noncompliance and dependent censoring in an AIDS clinical trial with inverse probability of censoring weighted (IPCW) log-rank tests. Biometrics. 2000;56(3):779–88.
Briefly summarise the key points from this paper.

Certainly — the **Robins & Finkelstein (2000)** paper is a foundational work that introduced the **IPCW log-rank test** to correct for **bias due to treatment noncompliance and dependent censoring** in randomised trials. Here's a structured summary of the **key points** relevant to your context:

🔑 Context and Motivation

- In clinical trials (e.g. AIDS, as in their motivating example), patients often **deviate from their assigned treatment**, either by discontinuing or switching treatments.
- When such **noncompliance or treatment switching is related to time-dependent covariates** (which also affect the outcome), **standard survival analyses (e.g., ITT or per-protocol) yield biased estimates**.

- The problem is one of **dependent (informative) censoring**, where censoring (due to treatment deviation) is **not independent of the outcome**.

🔧 Main Contribution

Robins & Finkelstein propose the **Inverse Probability of Censoring Weighting (IPCW) log-rank test**, which:

1. **Censors individuals at the time they deviate from their assigned treatment strategy** (e.g., when control patients switch to the experimental treatment).
2. **Weights each uncensored individual by the inverse of their probability of remaining uncensored**, conditional on their observed history up to that time.
3. This creates a **pseudo-population** in which **censoring is independent of potential outcomes**, under assumptions.

🧠 Key Assumptions

1. **Sequential randomisation assumption** (also called **no unmeasured confounding**):
   All variables that jointly influence treatment switching and survival must be **measured and correctly modelled**.
2. **Correct model specification**:
   The model used to estimate the censoring probabilities (often a Cox or logistic regression model) must be correctly specified.
3. **Positivity**:
   Every patient must have a **non-zero probability** of following their observed treatment trajectory, conditional on covariates.

📐 Methodological Details

- The **probability of remaining uncensored** at time t, given covariate history $\bar{L}_t$, is modelled (e.g., using Cox regression).
- The inverse of this probability is used as a **stabilised weight** to adjust the **risk sets** in a **weighted log-rank test** or **IPCW Cox model**.
- This adjustment allows unbiased estimation of the causal effect of a sustained treatment regime under a **hypothetical scenario** (e.g., "what if no one had switched?").

🧪 Application and Results

- The authors applied the method to an AIDS trial with noncompliance and showed that the IPCW log-rank test yielded **more valid estimates** than naive per-protocol or as-treated analyses.
- Simulation studies confirmed that the IPCW test maintained type I error and produced unbiased treatment effect estimates under correct assumptions.

📘 Implications for Your Study

- IPCW can be used to **estimate the causal effect of a sustained treatment strategy** in the presence of **treatment switching**, as in your trial.
- To apply IPCW in your case:
  - Define **switching as a censoring event**.
  - Model the **probability of not switching** at each time point, conditional on covariate history.
  - Use **inverse of this probability as a weight** in survival analysis (e.g., weighted log-rank or Cox model).
- This allows you to estimate the **hypothetical estimand** you defined earlier — OS under the assumption that no switching occurred.

Let me know if you'd like help with implementation in R, e.g., setting up the IPCW weights or fitting a weighted Cox model.

Could you elaborate more on the research question Robins and Finkelstein were trying to answer in their paper, mentioning the comparator arms, possible intercurrent events, outcome definition and population under study.

Certainly — here's a more detailed elaboration on the **research question** addressed by **Robins & Finkelstein (2000)** in their seminal paper on IPCW:

🔍 Research Question

> **What is the causal effect of early initiation of zidovudine (AZT) treatment versus delayed initiation on survival among patients with AIDS-related complex (ARC), had all patients adhered to their assigned treatment strategy?**

In other words: *"How would survival differ between early and deferred AZT treatment strategies if all patients had complied with their assigned regimen, without switching or noncompliance?"*

💊 Comparator Arms (Treatments)

- **Early treatment group (AZT initiated immediately)**:
  Patients were randomised to start AZT therapy at baseline.
- **Deferred treatment group (AZT initiated upon progression)**:
  Patients were randomised to begin AZT only when clinical progression occurred (e.g., based on CD4 count decline or clinical markers).

⚠️ Intercurrent Events (Post-randomisation Deviations)

The key intercurrent event was:

- **Noncompliance with assigned treatment** — specifically, **treatment switching** or **initiation of AZT in the deferred group before reaching the protocol-defined threshold**, or **discontinuation in the early group**.

These deviations introduced **informative censoring** because patients who switched or discontinued might have different survival prospects than those who adhered.

🎯 Outcome Definition

- **Primary outcome**:
  Time to death (all-cause mortality).

This was a time-to-event endpoint — a survival outcome — subject to right censoring due to dropout or study end, and **potentially dependent censoring due to treatment switching**.

👥 Population Under Study

- Patients enrolled in an **AIDS Clinical Trial**, specifically those with **AIDS-related complex (ARC)** — a symptomatic, pre-AIDS condition.
- All patients were **randomised** to early vs delayed AZT therapy, thus enabling a causal comparison under randomisation, **conditional on appropriate adjustment for post-randomisation deviations**.

🔄 Why IPCW Was Needed

The presence of **non-random treatment switching** meant that:

- **Intention-to-treat (ITT)** analysis would **underestimate the true benefit** of early treatment, since patients in the control arm often started AZT prematurely.
- **Per-protocol or as-treated analyses** would be **biased** due to **time-varying confounding**, since the decision to switch/start treatment depended on evolving patient health (e.g., CD4 counts, symptoms), which also affected mortality risk.

Thus, Robins and Finkelstein proposed IPCW as a way to **reconstruct a pseudo-population** in which patients are **censored at the time of switching**, but **weighted** so that the censoring is **independent of survival**, conditional on observed history.

✅ Goal

To estimate the **causal effect of sustained early versus delayed AZT treatment on mortality**, under the **hypothetical scenario** that all patients had adhered to their assigned strategy — *the very same estimand class you are targeting* in your lung cancer trial.

Would you like a sketch of how this relates structurally to your trial design in non-small cell lung cancer?

Attached, I provide you with a directed acyclic graph (DAG) that represents our research problem.
To simplify this DAG and take into consideration this deterministic information provided below, I restrict my DAG to the post-progression period for patients in the control arm only and consider two follow-up time points, 1 & 2.
Here A(k) is deterministically 0 if A(k-1)=0 AND L(k-1) (reflecting progression status)=0 (no progression).
Following disease progression, the decision as to whether or not a patient in the control group continues to take their treatment (A0 or A1) depends on predictive factors measured at or before the time of disease progression (L0).
Again, we assume that treatment intake (A0) affects outcome (Y) and influences a set of TVCS at a later time-point (L1).
These covariates are also assumed to impact the clinician's decision as to whether or not a patient should subsequently change treatments (A1), introducing time-varying confounding and treatment confounder feedback through L1.
I will emphasise again that in our case scenario, patients in the control group do not switch unless they progress, and all patients in the experimental group never switch.
I still want to estimate in my outcome model the causal effects of sustained treatment interventions (always taking control versus always taking experimental) under a hypothetical strategy where control patients do not switch treatment.
I want you to use this DAG as a basis for performing the appropriate IPCW analyses and provide me with the analysis R-script.
You may assume that the dataset is in long format, with variables: id, time (in months and increases with increments of 1), death indicator, switching indicator, baseline covariates
repeated over time points, time-varying covariates at each time pt.

Thank you — based on your detailed description and DAG structure, I'll walk you through the **IPCW setup** tailored to your scenario and then provide a customised **R script** for the analysis.

🎯 Goal Recap

You aim to estimate the **causal effect of sustained treatment with control vs. experimental** on **overall survival**, under a **hypothetical strategy** where **patients randomised to control never switch**, using **IPCW**.

🧠 Conceptual Setup

You restrict analysis to:

- **Patients in the control group**
- **Post-progression period**
- **Two time points (k = 1, 2)**

📌 Key causal features (from your DAG):

- $A_k \in \{0, 1\}$: Treatment status at time k; only control arm patients can switch.
- L_0: Baseline confounders (affect A_0, Y).
- L_1: Time-varying covariates (affected by A_0; affect A_1, Y).

- Y: Overall survival.
- **Switching (A = 1) is only possible after progression (L = 1).**
- Treatment-confounder feedback: $A_0 \to L_1 \to A_1 \to Y$

🔧 IPCW Implementation Logic

- **Artificial censoring**: You *censor* control patients at the time they **switch treatment**.
- **IPCW weights**: Estimate each patient's **probability of remaining uncensored (i.e., not switching)** up to time t, conditional on their observed covariate history.
- Use these weights in a **weighted Cox model** to estimate survival differences under a **hypothetical "no-switching" strategy**.

📦 Data Structure (long format assumed)

id	time	death	switched	A	L0_1	L0_2	L1_1	L1_2	...

- `A` = treatment at time `t` (binary, only switches from 0→1 in control arm)
- `switched` = 1 if patient has ever switched by time `t`, 0 otherwise
- `death` = 1 if patient died at time `t`, 0 otherwise
- `L0_*`, `L1_*` = baseline and time-varying covariates (e.g., ECOG, lesion size)

💻 R Script for IPCW Analysis

Here is an example R script using the `ipw` and `survival` packages:

```r
# Load required packages
library(survival)
library(ipw)
library(dplyr)

# STEP 1: Define censoring variable
# Censor at the time of switching
data <- data %>%
  group_by(id) %>%
  mutate(
    censored = ifelse(switched == 1 & A == 1, 1, 0),
    censored_time = ifelse(censored == 1, time, NA)
  )

# STEP 2: Estimate probability of remaining uncensored
# Use logistic regression for censoring model
censor_model <- glm(censored ~ time + L0_1 + L0_2 + L1_1 + L1_2,
                    data = data,
                    family = binomial())

# Predict censoring probabilities (i.e., staying uncensored)
data <- data %>%
  mutate(p_uncensored = 1 - predict(censor_model, type = "response"))

# STEP 3: Compute IPCW
data <- data %>%
  group_by(id) %>%
```

```
  mutate(ipcw_weight = cumprod(p_uncensored))

# Stabilised weights (optional):
# numerator model could include only baseline covariates
numerator_model <- glm(censored ~ L0_1 + L0_2,
                       data = data,
                       family = binomial())
data <- data %>%
  mutate(p_uncensored_numer = 1 - predict(numerator_model, type = "response")) %>%
  group_by(id) %>%
  mutate(ipcw_weight_stab = cumprod(p_uncensored_numer) / ipcw_weight)

# STEP 4: Fit weighted Cox model for survival (under "no switching")
# Keep only uncensored or pre-switching observations
analysis_data <- data %>% filter(censored == 0)

# Use stabilised weights (can also use unstabilised)
cox_model <- coxph(Surv(time, death) ~ A, data = analysis_data,
                   weights = ipcw_weight_stab, robust = TRUE)

summary(cox_model)
```

📌 Notes

- The **censoring model must include all predictors of switching and survival**, including time-varying confounders affected by prior treatment.
- You may consider **pooled logistic regression** or **discrete-time hazards models** for better time discretisation control.
- For **multiple time points or longer follow-up**, extend the structure accordingly and verify positivity and weight stability.

Would you like me to generate simulated data to test this script, help diagnose extreme weights, or adapt this to your actual covariate names and structure?
